# A dynamic Markov model to assess the cost-effectiveness of the Kidney Team at Home intervention in The Netherlands

**DOI:** 10.1007/s10198-021-01383-0

**Published:** 2021-10-13

**Authors:** Steef Redeker, Sohal Ismail, Hester V. Eeren, Emma K. Massey, Willem Weimar, Mark Oppe, Jan Busschbach, the Kidney Team at Home consortium

**Affiliations:** 1 Erasmus Medical Center, Section of Medical Psychology and Psychotherapy, Department of Psychiatry, Postal Address, PO Box 2040, 3000 CA Rotterdam, The Netherlands; 2 Erasmus MC, Transplant Institute, Department of Internal Medicine, Rotterdam, The Netherlands; 3 Maths in Health, Rotterdam, The Netherlands

**Keywords:** Patient education, Cost-effectiveness analysis, Organ donation, Home-based educational program, Markov model

## Abstract

**Objectives:**

The Kidney Team at Home program is an educational intervention aimed at patients with chronic kidney disease to assist them in their choice for kidney replacement therapy. Previous studies have shown that the intervention results in an increase in knowledge and communication on kidney replacement therapy, and eventually in an increase in the number of living donor kidney transplantations. The study assesses the cost-effectiveness of the intervention compared to standard care.

**Methods:**

A dynamic probabilistic Markov model was used to estimate the monetary and health benefits of the intervention in The Netherlands over 10 years. Data on costs and health-related quality of life were derived from the literature. Transition probabilities, prevalence, and incidence rates were calculated using a large national database. An optimistic and a pessimistic implementation scenario were compared to a base case scenario with standard care.

**Results:**

In both the optimistic and pessimistic scenario, the intervention is cost-effective and dominant compared to standard care: savings were €108,681,985 and €51,770,060 and the benefits were 1382 and 695 QALYs, respectively.

**Conclusions:**

The superior cost-effectiveness of the intervention is caused by the superior health effects and the reduction of costs associated with transplantation, and the relatively small incremental costs of the intervention. The favorable findings of this implementation project resulted in national uptake of the intervention in The Netherlands as of 2021. This is the first time a psychosocial intervention has been implemented as part of standard care in a kidney replacement therapy program worldwide.

## Introduction

Patients with chronic kidney disease (CKD) need kidney replacement therapy (KRT) to survive. There are four major types of KRT: hemodialysis (HD); peritoneal dialysis (PD); deceased donor kidney transplantation (DDKT); living donor kidney transplantation (LDKT).

Hemodialysis and peritoneal dialysis are associated with impaired quality of life and a high mortality. The median survival for patients on dialysis is 5 years [1], while the 5-year patient-survival after transplantation ranges from 86.1% to 95% [2]. Transplantation is the optimal treatment for most patients in terms of survival and quality of life. Because of a continuous scarcity of deceased donor kidneys, patients need to be on a wait list to be considered for deceased donor kidney transplantation. The average wait time for a kidney of a deceased donor in The Netherlands is 3.5 years, from the first day of dialysis [3]. For a living kidney donor transplantation, patients have to find a living kidney donor themselves.

A LDKT is the best treatment option in terms of quality of life and survival [4, 5]. However, there is inequality in access to LDKT for patients with CKD [6–8]. In particular, non-Western patients were less likely to undergo a LDKT [6, 9, 10]. To address this inequality, a home-based educational intervention has been developed in the United States, resulting in better knowledge on LDKT, an increase in the willingness to discuss LDKT with others, and an increase in LDKT-rates [11]. As a result of these findings, two randomized controlled trials (RCTs) testing the effectiveness of home-based educational interventions were conducted in The Netherlands [12, 13].

Despite differences in the patient population, these studies showed comparable results as the RCT in the USA: an increase in knowledge on all KRTs, better communication on LDKT, and an increase in LDKT-rates. These positive results led to an implementation project in which the Kidney Team at Home program was implemented on a larger scale at eight hospitals in four regions of The Netherlands [14]. The goal of this program was to assess whether the results of the previous studies could be replicated when the intervention was widely implemented in daily practice. This implementation project was conducted between 2016 and 2020. Replication of the RCT results would support nationwide deployment of the program as standard care. Demonstrating cost-effectiveness of the program could support further adoption of the program in standard care.

Augmenting standard care with the Kidney Team at Home program will increase costs and should be weighed against the health benefits of the program. Even more, because CKD is a costly disease. Per patient, the costs of dialysis in The Netherlands per year are between €80,000 and €120,000 and the cost of a single transplantation is around €80,000 [15]. As healthcare costs in The Netherlands are rising [16], there is a need to assess whether these additional costs are well spent. The present study, therefore, assesses the cost-effectiveness of the effects of the Kidney Team at Home intervention on the KRT-program compared to standard care.

Methods

### Markov model

The Markov model used for the cost-effectiveness analysis has a similar structure as the model from De Wit et al. (1998) [17]. A Markov modelling technique is applicable because the decision problem involves risk that is continuous over time, the timing of events is important, and events may happen more than once [18]. Within a Markov simulation, the time horizon of the study is divided into a number of discrete time periods, the so-called Markov cycles. A Markov process is based on the principle that patients are always in a certain disease state and that they can change between disease states once during each cycle. By assigning cost and effects to each disease state and keeping track of the time patients remained in each disease state, long-term cost and effects can be estimated.

**Fig. 1:**
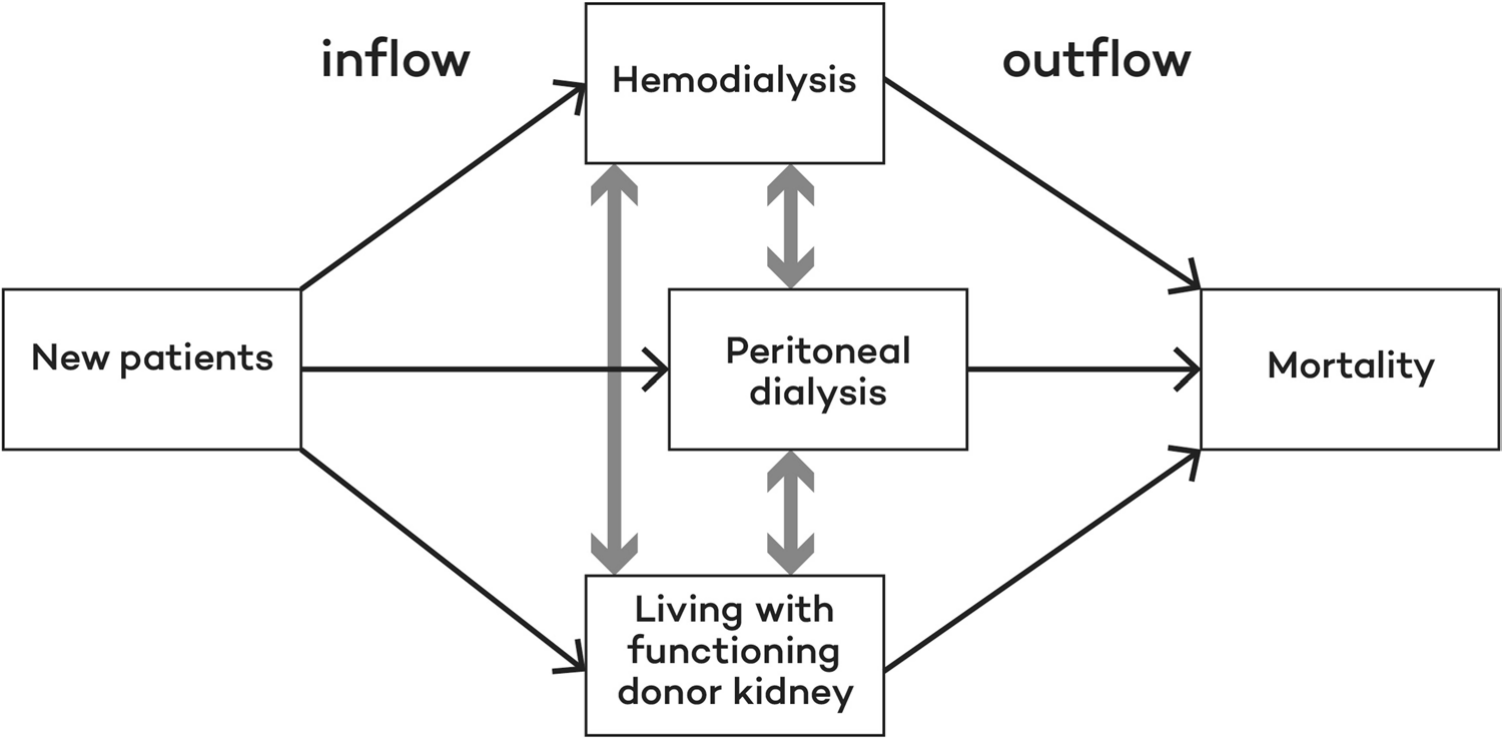
Simplified graphic representation of the Markov model

A simplified graphic representation of the Markov model showing only the treatment categories rather than all the individual Markov states is presented in Fig. 1. It is a so-called dynamic Markov model, as incident patients are added to the cohort and enter the model each cycle (inflow). The size of the inflow per month is based on epidemiological predictions as described in the paragraph “Incidence rates and prevalence”. After entering the model, patients can start on various treatment options. From there they can move between different treatment modalities until they die (outflow).

### Markov states

The Markov states were based on the treatments currently available in The Netherlands. These were: center hemodialysis (CHD), home hemodialysis (HHD), continuous ambulatory peritoneal dialysis (CAPD), continuous cyclic peritoneal dialysis (CCPD), deceased donor kidney transplantation (DDKT), and living donor kidney transplantation (LDKT). Dialysis treatment was divided into these four treatment modalities and transplantation into two since the expected transition probabilities may differ for each of them. Palliative care or conservative treatment were not included in the model, as it is not expected that the intervention will lead to a shift in the number of patients on these treatment options. Note that Fig. 1 is a simplified version of the model and does not show these refined differences in treatment modalities.

The transition probabilities between states in the model are not constant over time. The largest differences are between the first year and subsequent years. Therefore, we defined separate Markov states for the first year of treatment and for subsequent years of treatment within a specific modality. Incident patients that enter the model and prevalent patients that switch between treatment modalities are assigned to the first year Markov states, whereas patients that spend more than 1 year in any one Markov state are transferred to the subsequent year’s state of the same treatment modality. The cycle time of the model is one month, and the model was run for 120 cycles, i.e. 10 years.

#### Patient cohort

New patients flow into the model at the start of each cycle. These new patients are added to the number of patients that were already present in the model. These prevalent patients in the model represent the total Dutch CKD-population, as of January 1st 2018. Table 1 shows the demographic characteristics of that starting population, which is based on data of the Dutch Renal Registry ‘Renine’ [19]. Patients were divided into six different groups: age 0–44/45–64/65 + combined with being diabetic/non-diabetic.

**Table 1:** Baseline characteristics of prevalent patients on 1-1-2018

Baseline characteristics	Mean (SD)
Age	61.3 (15.7)
Male/Female (%)	61 / 39
Diabetes/Non-diabetes (%)	15 / 85
Treatment modality	***N*** ** = 16,917**
Center hemodialysis	5,516
Home hemodialysis	238
CAPD	402
CCPD	517
DDKT	4,817
LDKT	5,427

Parameters and data sources.

#### Costs

Costs for the various KRT treatments were retrieved from a Dutch study on the average annual healthcare costs for Dutch patients with a claim for dialysis or a kidney transplantation [15]. These costs include costs made by patients unrelated to KRT, such as costs related to complications or comorbidities. This cost study was based on the national database of all Dutch health insurance companies of the years 2012 to 2014 [20]. This detailed database holds the records of 99% of all Dutch citizens. The costs per KRT treatment are distinguished according to the following healthcare components: hospital care (in- and outpatient), primary care, mental health care, medication, medical devices, transportation, health care incurred abroad and claims of other types of health care. Hospital costs are subdivided in costs related to KRT, including costs of the dialysis procedure (including surgery for dialysis access), the kidney transplant (including donor expenses) as well as the preliminary and post-transplantation care. In the model, the costs per KRT treatment, and thus per Markov state in the model, were discounted at a rate of 4% per year which is in line with the Dutch guidelines for economic evaluations [21]. Intervention costs were estimated to be €2811 [22]. This estimation was made based on the implementation project with a micro-costing approach [14]. All baseline costs were expressed in 2018 Euros.

#### Outcomes (effects)

The effects were expressed as Quality Adjusted Life Years (QALYs). These ‘utilities’ used for this economic evaluation were obtained from a systematic review and meta-analysis by Liem et al. [23]. This systematic review extracted utilities from English studies that reported the EQ-5D, time-trade off and standard gamble values of CKD-patients. Mean utilities were calculated using random-effects models. Because of the preference of the Dutch health care institute for utilities estimated using the EQ-5D [21], the reported mean EQ-5D values and confidence intervals of the systematic review were used in the analysis. Since effects of cost–utility analyses are preferably expressed in Quality Adjusted Life Years (QALYs), QALYs were derived from these utility scores. In line with the Dutch guidelines QALYs were discounted at a rate of 1.5% per year [21].

#### Transition probabilities

The rate patient transition from one state to another, the transition probabilities, was calculated using empirical data from the Dutch Renal Registry ‘Renine’ [19]. The ‘Renine’ database contains information concerning all Dutch patients who undergo a non-experimental form of KRT. This database contains complete patient histories regarding treatment modalities, primary diagnosis, and background variables such as age for every CKD patient in The Netherlands. The first-year probabilities were based on data regarding the first 12 months of registration of the patients. The annual transition probabilities for subsequent years were based on the pooled annual event rates for the 2nd, 3rd and 4th years of the data.

#### Treatment incidence and prevalence

To estimate the incidence rates for the following ten years, parametric functions were estimated for each of the six patient groups (age 0–44/45–64/65 + and diabetic/non-diabetic) and the six treatment modalities. These 36 functions (6 mutually exclusive groups × 6 treatment options) were fitted using linear and non-linear regression analysis on the annual number of incident patients in the ‘Renine’ database from January 1st 2008 to December 31st 2017. The resulting predicted annual incidence rates were then transformed to monthly incidence rates to match the cycle time of the model. These monthly incidence rates represent the monthly inflow of new patients into the model. In some cases, there was insufficient data available to model incidence using regression techniques: i.e. in some cases almost none of the patients start KRT with a specific treatment, such as 65 + diabetic patients starting KRT with DDKT. In these cases the last observed value was carried forward. The baseline prevalence was the observed prevalence on January 1st 2018. In other words, the model uses the data on prevalence from January 1 2018 as the initial distribution of patients over the health states and age groups. An overview of all parameters is presented in Table 2.

**Table 2:** Parameter values and distributions

Dimension	Base case value	SE	Distr. For PSA
Health state utilities [23]			
CHD	0.56	0.033	Beta
HHD	0.56	0.033	Beta
CAPD	0.58	0.043	Beta
CCPD	0.58	0.043	Beta
DDKT	0.81	0.046	Beta
LDKT	0.81	0.046	Beta
Costs of intervention	€2811	281	Gamma
Therapy costs per year [15]			
Costs of CHD (1y-2y +)	€98,914	367	Gamma
Costs of HHD (1y-2y +)	€92,967	958	Gamma
Costs of CAPD (1y-2y +)	€82,824	1935	Gamma
Costs of CCPD (1y-2y +)	€96,043	1124	Gamma
Costs of DDKT 1y	€106,210	1009	Gamma
Costs of DDKT 2y +	€23,212	162	Gamma
Costs of LDKT 1y	€78,297	980	Gamma
Costs of LDKT 2y +	€22,716	149	Gamma

### Base case scenario

In the ‘base case scenario’, patients receive standard care: patients visiting the outpatient clinic receive standard education about the various modalities of KRT from a (transplant) nephrologist. Note that in this scenario, living donation is encouraged, as it is common that nephrologists ask if the patient has a living donor candidate in their social network and they will encourage the patient to discuss this with their loved ones. In 2017, 552 LDKTs were conducted in The Netherlands, and a parametric function based on the years 2008 to 2017 was used to estimate number of the LDKTs in 2018 and subsequent years.

### Kidney Team at Home scenario

Patients receiving the Kidney Team at Home intervention, receive two home visits: an intake and group education. The aim of the first home visit for the educators is to familiarize themselves with the social network of the patient and prepare for the second session. In the second session, the group educational intervention took place. The content and process of the intervention has been described elsewhere in more detail [24]. Patients who are eligible for the intervention are > 18 years of age and eligible for all KRT-options. Based on figures of the implementation study [25] we estimated that 35% of all incident patients would be eligible for the intervention. Results from the implementation project show that 42.5% of the eligible patients completed the group education. Of the patients who completed the intervention, approximately 18% underwent a LDKT within the 2-year follow-up and another 17% was in preparation for a LDKT. Approximately 2/3 of these patients underwent a LDKT as the first form of KRT, a pre-emptive transplantation. These parameters were used in the model to estimate the number of incident patients receiving the intervention and the effect size of the intervention.

In the Kidney Team at Home scenario, the effect of the intervention was evaluated in the model. Quality of life in terms of ‘utilities values’ and costs associated with the different treatment modalities (the Markov states) were not expected to differ between patients receiving the intervention and those who do not. Thus, for example, costs of dialysis remain the same, irrespective of receiving the Kidney Team at Home intervention. The cost of the intervention is added separately. In the model, the effects of the intervention are only expressed as an increase in LDKT, both pre-emptive LDKT (incident patients that come into the model in the LDKT health state) and LDKT after a period of dialysis. The impact of the intervention is therefore solely reflected in the incidence rates and transition probabilities. Transition probabilities and incidence rates to the other health states are proportionally lowered as the LDKT-rates increases. The effect of the intervention is built into the model after the first six cycles (6 months), which coincides with the moment the first effect was observed in the first RCT [12].

### Analysis

In addition to a deterministic analysis, the model includes probabilistic sensitivity analysis using Monte Carlo simulation. This means that not only the mean number of patients per year per treatment modality was estimated, but also the uncertainty surrounding those mean numbers of patients. Consequently, all model parameters (e.g. the transition probabilities) were used in the model as distributions rather than point estimates. In the Monte Carlo simulation, the model was evaluated a large number of times (5,000 times). For each evaluation, the model parameters were drawn from their distributions. This way all uncertainty from the model parameters was taken into account and reported in the results. The study was approved by the institutional review board of the Erasmus MC (MEC-2016–496).

Health utilities were assigned a beta distribution, costs were assigned a gamma distribution. Dirichlet distributions were estimated for all transition probabilities based on annual count data. For the effect size (number of extra transplantations as a result of the intervention) a standard error of 20% was assumed to account for uncertainty. For the intervention costs a standard error of 10% was used.

Compared to the base case scenario, two scenario analysis were conducted concerning the effect size. First, an optimistic scenario where we assume that 35% of the patients that received an intervention will end up undergoing a LDKT (scenario 1). Second, a pessimistic scenario where we assume that 18% of the patients undergoing a LDKT (scenario 2). These numbers were estimated based on the results of the implementation project [14].

Results of the cost-effectiveness analysis (optimistic scenario vs base case scenario, and pessimistic scenario vs base case scenario) were represented as incremental cost-effectiveness ratios (ICER).

### Data analysis

The model was implemented in Excel 2016 (Microsoft Corp., Redmond). IBM-SPSS Statistics version 25 (SPSS Inc., Chicago) was used to estimate the functions of the incidence rates.

## Results

Results of the deterministic and probabilistic sensitivity analysis scenarios after ten years are shown in Table 3. It shows that the Kidney Team at Home ‘dominates’ the base case (i.e., it results in more QALYs at lower costs) over a time horizon of 10 years.

**Table 3:** Deterministic and probabilistic results of the cost-effectiveness analyses

	Deterministic			Probabilistic		
Outcome	Costs	QALYs	ICER^a^	Costs (CI)	QALYs (CI)	ICER^a^
Basecase	€ 7,836,028,014	136,927		€7,881,810,446 (€7,503,847,711–€8,273,443,343)	135,960 (130,384—141,328)	
Scenario 1 (Optimistic)	€ 7,728,204,065	138,335	− 76,559	€7,773,128,461 (€7,404,698,547—€8,158,246,557)	137,341 (131,661—142,934)	− 78,666
Scenario 2 (Pessimistic)	€ 7,773,028,092	137,779	− 73,954	€7,830,803,911 (€7,456,069,881—€8,218,423,115)	136,630 (130,996—142,162)	− 74,498

^a^ Compared to basecase

Table 4 shows the predicted costs and QALYs of the base case scenario per year, which means that the second year is not a cumulative result of the first and second year, but only represents the costs and QALYs of all patients in the model in the second year. Table 5 and 6 represent the results of the optimistic and pessimistic scenario, respectively. The results show that the costs of the base case scenario are lower than the optimistic and pessimistic scenario in the first year, but are higher in subsequent years compared to both scenarios. The QALYs are higher for the Kidney Team at Home scenarios from the first year onwards.

**Table 4:** Discounted costs and QALYs Basecase

Base case	Costs		QALYs	
	Mean	[ 95% CI]	Mean	[ 95% CI]
12mo	€ 889,847,159	[€ 883,046,634; € 896,790,066]	12,169	[11,741; 12,553]
24mo	€ 878,195,028	[€ 861,623,929; € 895,772,638]	12,526	[12,083; 12,929]
36mo	€ 855,140,830	[€ 830,153,319; € 881,424,105]	12,865	[12,396; 13,301]
48mo	€ 828,799,465	[€ 796,411,726; € 862,720,881]	13,188	[12,686; 13,670]
60mo	€ 801,884,676	[€ 763,279,030; € 842,124,977]	13,497	[12,951; 14,019]
72mo	€ 775,347,290	[€ 731,413,386; € 821,316,907]	13,793	[13,207; 14,356]
84mo	€ 749,548,362	[€ 700,947,659; € 799,743,021]	14,078	[13,445; 14,689]
96mo	€ 724,639,297	[€ 672,367,167; € 778,997,109]	14,353	[13,672; 15,007]
108mo	€ 700,687,584	[€ 645,302,303; € 758,520,920]	14,618	[13,891; 15,319]
120mo	€ 677,720,755	[€ 619,649,947; € 738,505,236]	14,874	[14,101; 15,624]

**Table 5:** Discounted costs and QALYs for Scenario 1 (optimistic)

Optimistic	Costs		QALYs	
	Mean	[ 95% CI]	Mean	[ 95% CI]
12mo	€ 890,275,006	[€ 883,513,448; € 897,238,767]	12,172	[11,743; 12,557]
24mo	€ 875,710,486	[€ 859,323,557; € 892,900,803]	12,553	[12,107; 12,959]
36mo	€ 848,860,963	[€ 824,311,730; € 874,646,089]	12,919	[12,447; 13,364]
48mo	€ 819,139,714	[€ 787,581,230; € 852,575,480]	13,272	[12,755; 13,762]
60mo	€ 789,566,213	[€ 751,704,041; € 829,212,434]	13,613	[13,061; 14,152]
72mo	€ 761,109,269	[€ 718,245,901; € 805,813,440]	13,943	[13,345; 14,529]
84mo	€ 734,060,475	[€ 686,816,062; € 783,645,314]	14,262	[13,613; 14,892]
96mo	€ 708,479,670	[€ 657,445,420; € 761,379,425]	14,572	[13,868; 15,244]
108mo	€ 684,341,404	[€ 629,900,823; € 740,523,221]	14,872	[14,119; 15,595]
120mo	€ 661,585,261	[€ 604,605,948; € 720,289,001]	15,163	[14,363; 15,939]

**Table 6:** Discounted costs and QALYs for Scenario 2 (pessimistic)

Pessimistic	Costs		QALYs	
	Mean	[ 95% CI]	Mean	[ 95% CI]
12mo	€ 890,448,971	[€ 883,617,657; € 897,315,478]	12,170	[11,757; 12,565]
24mo	€ 877,319,806	[€ 860,732,312; € 894,275,424]	12,539	[12,104; 12,954]
36mo	€ 852,355,786	[€ 827,257,505; € 878,168,895]	12,891	[12,421; 13,343]
48mo	€ 824,317,626	[€ 792,049,124; € 857,697,413]	13,229	[12,725; 13,717]
60mo	€ 796,068,004	[€ 757,487,125; € 835,880,126]	13,553	[13,008; 14,089]
72mo	€ 768,563,807	[€ 724,792,614; € 813,911,486]	13,866	[13,277; 14,450]
84mo	€ 742,129,718	[€ 694,013,699; € 791,708,245]	14,168	[13,531; 14,797]
96mo	€ 716,870,513	[€ 664,759,902; € 770,435,770]	14,459	[13,772; 15,132]
108mo	€ 692,806,806	[€ 637,797,873; € 749,999,247]	14,741	[14,005; 15,462]
120mo	€ 669,922,874	[€ 612,277,467; € 730,267,126]	15,014	[14,225; 15,788]

The proportional magnitude of the uncertainty (expressed by the ratio of the range of the 95% CI and the estimates) is approximately the same for all scenarios with regard to the costs and the QALYs. In the final year, the magnitude of the uncertainty for the base case, optimistic and pessimistic scenario is 10% for the costs and 8% for the QALYs.

**Fig. 2:**
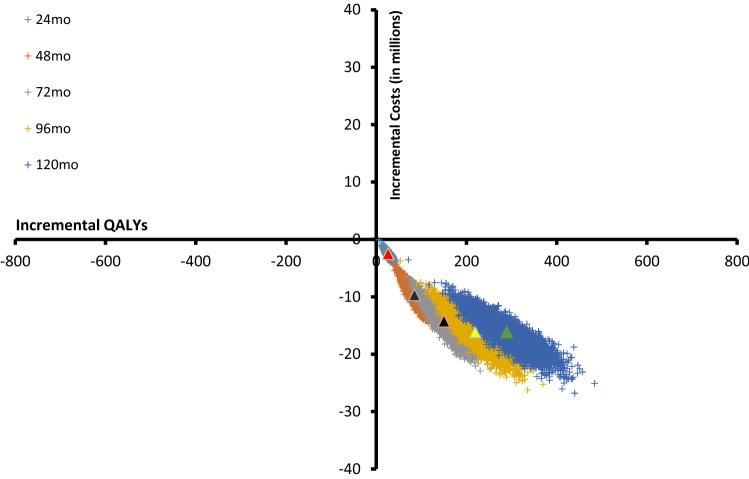
Graphic representation of the mean difference in incremental costs and incremental effects when comparing base case scenario to the optimistic KTAH scenario using 5000 simulations

**Incremental cost-effectiveness ratios:** The deterministic ICERs are € -76.559 and € -73.954 for the base case compared to the optimistic and pessimistic scenario, respectively. The following Tables (7, 8) present the probabilistic ICERs per year while comparing the base case to the Kidney Team at Home scenarios. In line with the costs and effects of Table 4, 5, 6, Table 7 shows that, in both the optimistic scenario and the pessimistic scenario, the first year yields additional costs and approximately the same number of QALYs. In the subsequent years, not only does the intervention results in πmore QALYs, but also produces monetary benefits, resulting in negative values for the ICERs. Figure 2 shows the cost-effectiveness plane of the base case compared to the optimistic scenario for the different years, which all appear at the bottom-right quadrant of the figure indicating dominance. The crosses represent point estimates of the ICERs and expand as time increases due to increasing uncertainty. The triangles represent the mean ICERs of the particular year.

**Table 7:** Incremental cost-effectiveness ratio. Base case versus scenario 1 (optimistic)

	Base case vs Optimistic		
	∆ Cost	∆ QALY	ICER
12mo	€ 427,847	3	122,649
24mo	€ − 2,484,542	26	− 94,584
36mo	€ − 6,279,867	54	− 115,633
48mo	€ − 9,659,752	85	− 114,179
60mo	€ − 12,318,463	117	− 105,617
72mo	€ − 14,238,020	150	− 94,942
84mo	€ − 15,487,887	184	− 84,077
96mo	€ − 16,159,627	219	− 73,783
108mo	€ − 16,346,180	254	− 64,341
120mo	€ − 16,135,495	289	− 55,829
Totaal	€ − 108,681,985	1,382	− 78,666

**Table 8:** Incremental cost-effectiveness ratio. Base case versus Scenario 2 (pessimistic)

	Base case vs Pessimistic		
	∆ Cost	∆ QALY	ICER
12mo	€ 601,628	2	344,565
24mo	€ − 882,334	13	− 66,787
36mo	€ − 2,811,232	27	− 102,873
48mo	€ − 4,531,139	43	− 106,425
60mo	€ − 5,889,038	59	− 100,334
72mo	€ − 6,876,749	75	− 91,132
84mo	€ − 7,529,722	93	− 81,249
96mo	€ − 7,894,182	110	− 71,660
108mo	€ − 8,016,672	128	− 62,749
120mo	€ − 7,940,620	145	− 54,645
Totaal	€ − 51,770,060	695	− 74,498

## Conclusions

The Kidney Team at Home has been shown to be highly cost-effective compared to standard care. This is caused by the superior effects and the reduction of costs associated with transplantation, which is propelled by the intervention at small incremental costs. This is caused by the superior health effects and the reduction of costs associated with transplantation, and the relatively small incremental costs of the intervention. The favorable results of this analysis resulted in national uptake of the intervention in standard care. To our knowledge, this is the first time a psychosocial intervention has been implemented as part of standard care in a kidney replacement therapy program worldwide.

## Discussion

The present study shows that the Kidney Team at Home intervention is cost-effective compared to standard-care. The results show that both Kidney Team at Home scenarios are superior to standard-care: the intervention ‘dominates’ standard-care with a higher number of QALYs and lower costs. From the first year onwards, the intervention saves costs while gaining health in terms of quality of life. In The Netherlands, with approximately 17.4 million inhabitants, the optimistic scenario and pessimistic scenario predict a cost saving of €107,823,949 and €62,999,923, respectively, and a gain of 1,408 QALYs and 852 QALYs over the next 10 years for the total KRT program, compared to standard care.

Our findings are consistent with other studies concerning the cost-effectiveness of KRT programs [17, 26, 27]. These studies found a large increase in health effects while saving costs when the number of LDKTs increased. We showed that our home-based educational program is a cost-effective strategy to educate patients and reduce the inequality in access to LDKTs. The incremental costs of the intervention are small compared to the costs of the treatment modalities. For instance, the cost per intervention is about 3% of the cost of 1-year dialysis.

The effectiveness of the intervention (i.e. the number of additional LDKTs due to the Kidney Team at Home intervention) impacts the ICER, as is seen in the difference in QALYs between the optimistic and pessimistic scenario in Tables 5 and 6. It is, therefore, important to safeguard the quality and hence the effects of the intervention. Indeed, a lot attention was given to maintaining the quality of the intervention during the implementation project, by having regular supervision, peer-to-peer meetings and training sessions. The costs of this quality assurance were also included in the costs of the intervention. Given that the influence of the costs on the ICER is less than the effect of the quality of the intervention, it seems justified to support this quality assurance system.

## Implications

Our findings suggest that the demand for the various treatment options for CKD may change. More LDKTs may lead to a drop of the number of patients needing dialysis. This might influence the demand for dialysis centers and transplantation facilities. Furthermore, it can be expected that patients who are unable to find a living donor in the program, will nevertheless profit from an increase in the number of LDKTs, as the total demand for deceased donor kidney reduces. The increase in living donation may lower the time spent on the waiting list for a DDKT. Future research should investigate whether it is indeed possible to reduce the number of dialysis centers and their associated costs.

Our cost effectiveness results support the request for national funding of the Kidney Team at Home. The favorable results of this analysis resulted in national uptake of the intervention in standard care in The Netherlands. There is little reason to think that the conclusions from this study will be much different for other countries with a developed health care system, as the effectiveness of home-based education has been demonstrated elsewhere and previously conducted cost-effectiveness analyses of KRT programs. Some countries are already investigating the implementation of home-based education, most notably in the UK were several initiatives have been undertaken to address disparities in access to LDKT. The results of these single-center studies were favorable. We therefore recommend that other countries should also investigate implementation of the Kidney Team at Home intervention.

## Strengths and limitations

There are certain strengths of our study. First, the parameters were derived from large datasets. The transition probabilities and incidence rates were calculated using the database of the Dutch Renal Registry ‘Renine’, containing virtually all patients receiving KRT between 2000 and 2018. The costs of the different health states were based on a study that used claims data of adult patients with at least one health insurance claim related to KRT in the period 2012–2014. The authors of this cost study stated that the number of incident and prevalent patients on KRT identified by the claims data were comparable to those identified by ‘Renine’, indicating that there is an overlap in patient population used for calculating the costs and transition probabilities. Because these parameters were based on large datasets, the uncertainty surrounding the ICERs is relatively small, as seen in the cost-effectiveness plane. This relative small degree of uncertainty is also the reason why we did not present one-way sensitivity analyses. The standard errors of the point estimates were relatively low (see: Table 2), which resulted in a minimal range of the ICERs. We therefore decided not to include the results of these analyses in this study.

There are also limitations of our study. First, the utility values were derived from the literature published in 2008 [23]. Another systematic review and meta-analysis was also available [28], however, this study only incorporated one study that used the EQ-5D among patients living with a kidney transplant. Most of the utility values reported in the study were derived from the SF-36, and they had to impute standard deviations, which may have affected their results [28]. Therefore, the systematic review of Liem et al. seemed the better choice for this economic evaluation [23].

Second, some parameters were derived from the implementation project, such as the percentage of patients who are willing to receive the home-based intervention (42.5%). If implemented in standard care, this percentage will likely be higher as the participation rate in studies are often lower than in the real-life setting, partly because of questionnaires and informed consent forms which were part of the implementation project [29]. In other words, the used participation rate may be an underestimation, which suggests that the ICER is even more favorable.

Finally, one needs to keep in mind that the accuracy of the model predictions depends on the accuracy of the transition probabilities and the model structure. Therefore, unexpected shifts in the trends of transition probabilities can have a profound impact on the accuracy of the forecasts by the model. For example, a change to the donor registration or allocation system (such as from opt-out to opt-in), could increase the number of deceased donor kidneys substantially [30]. This could have an effect on the willingness to donate a living donor kidney, as the urgency to ‘save’ a patient from dialysis may reduce. Consequently, the transition probabilities would change for and to every health state. In addition, the consequences of the COVID-19 pandemic have a large influence on the care of CKD-patients. Hence, the transplantation program had been temporarily put on hold, and at the time of writing, the number of transplantation is still significant lower than before the pandemic [31, 32].

### Next steps

The model was designed in such a way that it can easily be extended to allow for different endpoints. For example, updating the number of available deceased donor kidneys due to the new Dutch opt-out donor law, will allow the assessment of predicted prevalence of this policy change.

## Data Availability

The data that support the findings of this study are available on request from the corresponding author. The data are not publicly available due to privacy or ethical restrictions.

## References

[1] Naylor KL, Kim SJ, McArthur E (2019). Mortality in incident maintenance dialysis patients versus incident solid organ cancer patients: a population-based cohort. Am J Kidney Dis,.

[2] Wang JH, Skeans MA, Israni AK (2016). Current status of kidney transplant outcomes: dying to survive. Adv Chronic Kidney Dis..

[3] Stichting NT. Wanneer ben ik aan de beurt voor een niertransplantatie? : Nederlandse Transplantatie Stichting, 2019.

[4] Mahillo B, Carmona M, Alvarez M (2011). 2009 global data in organ donation and transplantation: activities, laws, and organization. Transplantation.

[5] Kramer A, Pippias M, Noordzij M (2018). The european renal association - european dialysis and transplant association (ERA-EDTA) registry annual report 2015: a summary. Clin Kidney J..

[6] Roodnat JI, van de Wetering J, Zuidema W (2010). Ethnically diverse populations and their participation in living kidney donation programs. Transplantation.

[7] Taylor DM, Bradley JA, Bradley C (2019). Limited health literacy is associated with reduced access to kidney transplantation. Kidney Int..

[8] Oniscu GC, Ravanan R, Wu D (2016). Access to Transplantation and Transplant Outcome Measures (ATTOM): study protocol of a UK wide, in-depth, prospective cohort analysis. BMJ Open.

[9] Roodnat JI, Laging M, Massey EK (2012). Accumulation of unfavorable clinical and socioeconomic factors precludes living donor kidney transplantation. Transplantation.

[10] Ismail SY, Claassens L, Luchtenburg AE (2013). Living donor kidney transplantation among ethnic minorities in The Netherlands: a model for breaking the hurdles. Patient Educ Couns..

[11] Rodrigue JR, Cornell DL, Lin JK (2007). Increasing live donor kidney transplantation: a randomized controlled trial of a home-based educational intervention. Am J Transplant..

[12] Ismail SY, Luchtenburg AE, Timman R (2014). Home-based family intervention increases knowledge, communication and living donation rates: a randomized controlled trial. Am. J. Transplant..

[13] Massey EK, Gregoor PJ, Nette RW (2016). Early home-based group education to support informed decision-making among patients with end-stage renal disease: a multi-centre randomized controlled trial. Nephrol Dial. Transplant.

[14] Redeker S, Oppe M, Visser M (2019). Cost-effectiveness of a home-based group educational programme on renal replacement therapies: a study protocol. BMJ Open.

[15] Mohnen SM, van Oosten MJM, Los J (2019). Healthcare costs of patients on different renal replacement modalities - Analysis of Dutch health insurance claims data. PLoS ONE.

[16] CBS. Zorguitgaven; kerncijfers. 2018.

[17] de Wit GA, Ramsteijn PG, de Charro FT (1998). Economic evaluation of end stage renal disease treatment. Health Policy.

[18] Briggs A, Sculpher M (1998). An introduction to Markov modelling for economic evaluation. Pharmacoeconomics.

[19] Nefrovisie. Renine. Nefrovisie, 2013.

[20] de Boo A (2011). Vektis - information center for health care services (In Dutch: Vektis 'Informatiecentrum voor de zorg'). TSG - Tijdschrift voor gezondheidswetenschappen..

[21] Nederland Z. Richtlijn voor het uitvoeren van economische evaluaties in de gezondheidszorg. Diemen: Nederland Zorginstituut 2015.

[22] Redeker S, Ismail SY, Massey EK, et al. Eindrapportage project 'Nierteam aan Huis' 2016–2020. 2020.

[23] Liem YS, Bosch JL, Hunink MG (2008). Preference-based quality of life of patients on renal replacement therapy: a systematic review and meta-analysis. Value Health.

[24] Ismail SY, Luchtenburg AE, Zuidema WC (2012). Multisystemic engagement and nephrology based educational intervention: a randomized controlled trial protocol on the KidneyTteam At Home study. BMC Nephrol.

[25] Redeker S, Ismail SY, Busschbach JJV, et al. NATIONAL IMPLEMENTATION OF THE KIDNEY TEAM AT HOME EDUCATIONAL INTERVENTION. Transplantation. 2020; 104.

[26] Haller M, Gutjahr G, Kramar R (2011). Cost-effectiveness analysis of renal replacement therapy in Austria. Nephrol Dial. Transplant.

[27] Howard K, Salkeld G, White S (2009). The cost-effectiveness of increasing kidney transplantation and home-based dialysis. Nephrology (Carlton).

[28] Wyld M, Morton RL, Hayen A, et al. A systematic review and meta-analysis of utility-based quality of life in chronic kidney disease treatments. PLoS Med. 2012; 9: e1001307. 10.1371/journal.pmed.1001307 PMC3439392 22984353

[29] Knottnerus JA, Tugwell P (2016). Prevention of premature trial discontinuation: how to counter Lasagna's law. J Clin Epidemiol..

[30] Madden S, Collett D, Walton P (2020). The effect on consent rates for deceased organ donation in Wales after the introduction of an opt-out system. Anaesthesia.

[31] Stichting NT. Verlossende donatie bij leven uitgesteld door corona. NTS, 2020.

[32] de Vries APJ, Alwayn IPJ, Hoek RAS (2020). Immediate impact of COVID-19 on transplant activity in The Netherlands. Transpl Immunol.

